# Stress‐induced modulation of volume‐regulated anions channels in human alveolar carcinoma cells

**DOI:** 10.14814/phy2.13869

**Published:** 2018-10-14

**Authors:** Martin D. Bach, Belinda H. Sørensen, Ian H. Lambert

**Affiliations:** ^1^ Section of Cell Biology and Physiology Department of Biology University of Copenhagen Copenhagen Ø Denmark

**Keywords:** Akt, cisplatin, LRRC8A, mTORC2, p53, reactive oxygen species

## Abstract

Shift in the cellular homeostasis of the organic osmolyte taurine has been associated with dysregulation of the volume‐regulated anion channel (VRAC) complex, which comprises leucine‐rich repeat‐containing family 8 members (LRRC8A‐E). Using SDS‐PAGE, western blotting, qRT‐PCR, and tracer technique ([^3^H]taurine) we demonstrate that reactive oxygen species (ROS) and the cell growth‐associated kinases Akt/mTOR, play a role in the regulation of VRAC in human alveolar cancer (A549) cells. LRRC8A is indispensable for VRAC activity and long‐term exposure to hypoosmotic challenges and/or ROS impairs VRAC activity, not through reduction in total LRRC8A expression or LRRC8A availability in the plasma membrane, but through oxidation/inactivation of kinases/phosphatases that control VRAC activity once it has been instigated. Pursuing Akt signaling via the serine/threonine kinase mTOR, using mTORC1 inhibition (rapamycin) and mTORC2 obstruction (Rictor knockdown), we demonstrate that interference with the PI3K‐mTORC2‐Akt signaling‐axes obstructs stress‐induced taurine release. Furthermore, we show that an increased LRRC8A expression, following exposure to cisplatin, ROS, phosphatase/lipoxygenase inhibitors, and antagonist of CysLT1‐receptors, correlates an increased activation of the proapoptotic transcription factor p53. It is suggested that an increase in LRRC8A protein expression could be taken as an indicator for cell stress and limitation in VRAC activity.

## Introduction

### Volume‐sensitive organic osmolyte release

Mammalian cells counteract volume changes, following osmotic perturbation, by accumulating / releasing osmolytes (Hoffmann et al. 2009; Lambert et al. 2015b). A reduction in cell volume following osmotic cell swelling (regulatory volume decrease, RVD) or as part of the apoptotic cell death process (apoptotic volume decrease, AVD) will in most mammalian cells imply net loss of inorganic ions (K^+^, Cl^−^) via volume‐sensitive ion channels as well as net loss of amino acids via a volume‐sensitive leak pathway for organic osmolytes (Lang 2007; Hoffmann et al. 2009). Furthermore, cells have the ability to shift the cell volume to instigate cell proliferation, migration, and apoptosis (Lang 2007; Hoffmann et al. 2009). Today it is assumed that volume‐regulated anion channel (VRAC) complexes, composed of members of the leucine‐rich repeat‐containing protein 8 family (LRRC8A‐E) (Planells‐Cases et al. 2015; Stauber 2015; Schober et al. 2017), are responsible for the volume‐sensitive chloride current (*I*_Cl, swell_) as well as swelling‐induced release of neurotransmitters and organic osmolytes (e.g., GABA, aspartate, glutamate, and taurine) (Qiu et al. 2014; Voss et al. 2014; Mongin 2016; Syeda et al. 2016; Lutter et al. 2017).

Each LRRC8 subunit consists of four transmembrane helix domains with a cytosolic C‐terminal containing up to 17 leucine‐rich repeat‐domains. Co‐immunoprecipitation in HEK cells shows that LRRC8A interacts with other LRRC8 subunits, forming a heteromeric, channel complex (Voss et al. 2014; Planells‐Cases et al. 2015; Stauber 2015; Schober et al. 2017). LRRC8A has been shown to be indispensable for VRAC activity (Qiu et al. 2014; Voss et al. 2014). Knockdown (KD) of the LRRC8D subunit disrupts taurine, *myo*‐inositol and GABA efflux during swelling‐induced RVD, albeit not to the same degree as KD of LRRC8A (Planells‐Cases et al. 2015; Lutter et al. 2017). LRRC8D KD has apparently no effect on *I*_Cl, swell_ (Planells‐Cases et al. 2015; Lutter et al. 2017). Recently, Mongin and coworkers demonstrated that LRRC8D KD in rat astrocytes reduced the swelling‐induced efflux of [^3^H]taurine by 50%, abolished *myo*‐[^3^H]inositol efflux but had no effect on D‐[^14^C]aspartate efflux (Schober et al. 2017). It was also demonstrated that the effect of LRRC8D KD on taurine release was lifted when extracellular pH was shifted from a physiological pH 7.4 (≈ 4% taurine negatively charged), to pH 9.8 (≈ 91% taurine negatively charged) (Schober et al. 2017). This provides evidence that a LRRC8A/D containing VRAC complex favors transports of the electroneutral, organic osmolytes.

Attempts have been made to illustrate the correlation between LRRC8A protein expression and VRAC activity. Exposure to protolichesterinic acid, derived from lichens, has previously been demonstrated to reduce LRRC8A expression and concomitantly volume‐sensitive taurine release in human alveolar carcinoma (A549) cells (Thorsteinsdottir et al. 2016). Furthermore, a reduced taurine release following hypoosmotic perturbation from cisplatin‐sensitive A549 cells correlated with a reduced expression of LRRC8A in the plasma membrane (Sørensen et al. 2016b). However, overexpression of MYC‐ or GFP‐tagged LRRC8A plasmids, resulted in a decrease in *I*_Cl,Swell_ in HEK293 and HeLa cells although the exogenous protein via immunocytochemistry was shown to reach the plasma membrane (Lee et al. 2007; Qiu et al. 2014). It appears that posttranslational modulation of LRRC8 proteins or modulators of the channel complex VRAC dictates activity.

### Volume‐sensitive taurine release – Reactive oxygen species (ROS)

The cell signaling pathway, activated by osmotic cell swelling and leading to release of taurine has been characterized in various cell types. In the case of Ehrlich ascites tumor cells and NIH3T3 mouse fibroblasts activation of volume‐sensitive taurine release involves phospholipase A_2_ (PLA_2_) mediated mobilization of arachidonic acid (AA) from phospholipids (the PLA_2_ subtype is cell type‐specific), 5‐lipoxygenase (5‐LO)‐mediated oxidation of AA to leukotrienes, and subsequently a cysteine‐leukotriene‐receptor (CysLT1)‐dependent activation of the taurine release system (Lambert et al. 2015b). In the A549 cell line it has likewise been demonstrated that 5‐LO and CysLT1 are essential components for taurine release during RVD (Holm et al. 2013). Once activated, the volume‐sensitive taurine release can be boosted by exposure to ROS, and through Ca^2+^ mobilization (Holm et al. 2013; Lambert et al. 2015b). ROS are produced within minutes following osmotic cell swelling (Lambert 2003; Ørtenblad et al. 2003; Varela et al. 2004; Diaz‐Elizondo et al. 2006; Friis et al. 2008; Holm et al. 2013) and it has been shown that the NADPH‐oxidase NOX4 contributes to swelling‐induced ROS production in NIH3T3 (Lambert 2003; Friis et al. 2008) and A549 (Holm et al. 2013) cells. Similarly, NOX4 and NOX2 have been demonstrated to be involved in swelling‐induced ROS production in hepatoma cells (Varela et al. 2004) and rabbit atrial/ventricular myocytes (Deng et al. 2010). An increased ROS accumulation, simulated by addition of hydrogen peroxide (H_2_O_2_) or inhibition of antioxidative enzymes (e.g., catalase), has accordingly been shown to potentiate swelling‐induced taurine release (Lambert 2003, 2007; Lambert et al. 2014). The effect of ROS on the volume‐sensitive taurine release is mimicked by the tyrosine phosphatase inhibitor sodium orthovanadate, indicating that ROS block tyrosine phosphatases presumably through oxidation of cysteines in their catalytic center (Meng et al. 2002). It is assumed that increased net tyrosine‐phosphorylation of elements in the volume‐sensitive signaling cascade and/or members of the LRRC8 family, boosts the transport efficiency of the VRAC complex (Lambert et al. 2015b). In this context, it is noted that proteomic studies reveals that LRRC8 proteins contains an array of sites available for posttranslational modification (phosphorylation, acetylation, ubiquitination, and N‐linked glycosylation) (Abascal and Zardoya 2012) and that LRRC8A/LRRC8E heteromeric channels, when exogenously expressed in *Xenopus* oocyte, are activated by oxidation, whereas exogenously expressed LRRC8A/LRRC8C as well as LRRC8A/LRRC8D channels are inhibited by oxidation (Gradogna et al. 2017).

### Volume‐sensitive taurine release – Akt‐mTOR signaling

The serine/threonine kinases Akt (protein kinase B) and mTOR have been demonstrated to modulate swelling‐induced taurine release (Lezama et al. 2005; Holm et al. 2013; Lambert et al. 2015b). In cultured cerebellar granule neurons, cell swelling stimulates Akt, whereas inhibition of kinases upstream to Akt (ErbB4, FAK, Src, and PI3K (Phosphatidylinositol‐4,5‐bisphosphate 3‐kinase)) suppresses Akt activation and reduces the concomitant release of taurine (Lezama et al. 2005). Akt activation, which is often associated with cell proliferation, growth, survival, metabolism, and autophagy (Manning and Toker 2017), implies recruitment of Akt to phosphatidylinsitol‐3,4,5‐triphosphate (PIP_3_) in the inner leaflet of the plasma membrane and a subsequent sequential phosphorylation of Akt at Thr‐308 and Ser‐473 (Hay 2005). Akt activity is indirectly reversed by the tumor suppressor PTEN (phosphatase and tensin homolog), a phosphatase that antagonizes PI3K activity by dephosphorylation of PIP_3_. It is the phosphoinositide‐dependent kinase‐1 (PDK1) that is responsible for phosphorylation of Akt at Thr‐308, whereas phosphorylation of Ser‐473 on Akt requires activation of mammalian target of rapamycin (mTOR). mTOR represent the catalytic subunit of two distinct complexes; mTORC1 and mTORC2, where mTORC1 operates down‐stream to Akt and mTORC2, which is typically activated by extracellular stimuli such as growth factors and insulin in a PI3K‐dependent manner (Liu et al. 2015; Manning and Toker 2017), activates Akt. mTORC2 is, in contrast to mTORC1, insensitive to rapamycin inhibition (Bai et al. 2017). In this context it has been suggested that PIP_3_, besides PDK1 and Akt, also recruits SIN1 to the plasma membrane where SIN1, through a conformational change in the mTORC2 complex, relives auto‐inhibition of mTOR and hence ensures mTOR kinase activity (Manning and Toker 2017). We have previously shown that mTORC1 activity is significantly increased within minutes following osmotic cell swelling but reduced following prolonged hypotonic treatment (Lambert et al. 2014).

### LRRC8A protein expression and p53‐mediated signaling to apoptosis

Apoptosis is a well‐orchestrated cell death program, characterized by chromatin condensation, membrane budding, phosphatidylserine (PS) externalization to the outer leaflet of the plasma membrane, cell shrinkage, and intracellular protein degradation due to activation of caspases. Kinases, triggered by reversible DNA damage, activate the check‐point kinase 2, which subsequently phosphorylates the tumor suppressor p53 (Roos and Kaina 2013). p53 orchestrates expression of genes involved in DNA repair, cell cycle arrest, and apoptosis (Hientz et al. 2017). In the latter case, this includes proapoptotic members of the Bcl‐2 protein family, for example, PUMA (p53‐upregulated modulator of apoptosis) and BAX (Bcl‐2‐associated X protein). PUMA and BAX facilitate mitochondrial cytochrome‐c release, activation of caspase‐9 through interaction with APAF‐1 (apoptotic protease‐activating factor), and finally activation of executioner caspases (caspases 3, 6, and 7) (Dasari and Tchounwou 2014; Mehmood 2014). Phosphorylation and activation of p53 is known to follow hyperosmotic cell shrinkage and exposure to Pt‐based chemotherapeutic drugs, for example, cisplatin (Friis et al. 2005; Lambert et al. 2015a; Sørensen et al. 2016b) and the subsequent activation of apoptotis is clearly dependent on LRRC8A expression/VRAC activity (Hoffmann and Lambert 2014; Planells‐Cases et al. 2015; Sørensen et al. 2016a). Originally it was assumed that resistance to cisplatin reflected limitation in osmolyte loss due to impairment of the activity of volume‐sensitive osmolyte transporters (Poulsen et al. 2010). However, more recently it has been demonstrated that cisplatin resistance correlated with limitation in cisplatin uptake and consequently annulation of the intracellular, cisplatin‐induced apoptotic cell death signaling (Planells‐Cases et al. 2015; Sørensen et al. 2016a). As cisplatin uptake in cisplatin‐sensitive A2780 cells is reduced by pharmacological inhibition of VRAC and by LRRC8A KD (Sørensen et al. 2016a) it is assumed that any stress‐induced modulation of LRRC8A expression/ VRAC activity will impact cisplatin sensitivity through limitation of drug uptake and hence impairment of the instigation of AVD and intracellular apoptotic cell signaling.

With emphasis on acute as well as long‐term exposure to ROS (H_2_O_2_), cisplatin and adaptation to hypotonic media we have used the A549 cell line to investigate the correlation between cell stress, total LRRC8A mRNA/protein expression, LRRC8A protein expression in the plasma membrane and VRAC activity. It will be demonstrated that prolonged cell stress reduce VRAC activity – not through reduction in total expression/plasma membrane availability of the LRRC8A protein but more likely through shift in the activity of kinases/phosphatases involved in volume‐sensitive cell signaling that ensures VRAC activity. It is suggested that an increased LRRC8A protein expression indicates cell stress as it correlates with activation of the proapoptotic transcription factor p53, following exposure to cisplatin, ROS, and pharmacological inhibitors of key elements, involved in release of organic osmolytes (taurine).

## Materials and Methods

### Chemicals and antibodies

Antibiotics (penicillin, streptomycin), Roswell Park Memorial Institute (RPMI‐1640) and Dulbecco's Modified Eagle (DMEM) media, fetal calf serum (FBS), and trypsin/EDTA were from Invitrogen, Denmark. [1,2‐^3^H(N)]‐taurine (NET1205250UC, specific activity: 0.707 TBq/mmol) and scintillation cocktail (Ultima Gold™) were from Perkin Elmer, Denmark. Unless otherwise stated, chemicals were purchased from Sigma Aldrich (St. Louis, MO) or Calbiochem (Europe). The following stock solutions were prepared: ETH 615‐139 (donated by Dr. I Ahnfelt‐Rønne, Løvens Kemiske Fabrik, Denmark, 2 mmol L^−1^, EtOH); bpV(HOpic) (Bisperoxo(bipyridine) oxovanadium, 200 *μ*mol L^−1^, ddH_2_O); MG132 (carbobenzoxy‐Leu‐Leu‐leucinal, 10 mmol L^−1^, DMSO); Vanadate (Sodium orthovanadate, 20 mmol L^−1^, ddH_2_O); Wortmannin (1 mmol L^−1^, DMSO); Rapamycin (400 *μ*mol L^−1^, EtOH); BHT (Butylated hydroxytoluene, 400 mmol L^−1^, EtOH); Trypsin‐EDTA solution (Invitrogen, 5 mg porcine trypsin, 2 mg EDTA pr. mL PBS); Zafirlukast (N‐[3‐[[2‐Methoxy‐4‐[[[(2‐methylphenyl) sulfonyl]amino]carbonyl]phenyl]methyl]‐1‐methyl‐ 1H‐indol‐5‐yl] carbamic acid cyclopentyl ester, 30 mmol L^−1^, DMSO).

#### Primary antibodies

Anti‐*β*‐actin (A1978, mouse monoclonal Ab, 42 kDa, Sigma‐Aldrich; 1:1000); Anti‐Akt // Anti‐Phospho‐Akt (9272 // 4058, rabbit Ab, 60 kDa, Cell Signaling, Danvers, MA; 1:500 // 1:250 (P‐Akt)); Anti‐histone H3 (9717, rabbit Ab, 18 kDa, Cell Signaling 1:250); Anti‐human‐LRRC8A (SAB1412855, mouse monoclonal Ab, 95 kDa, Sigma‐Aldrich; 1:250); Anti‐FoxO3a // Anti‐Phospho‐FoxO3 (Ser413) (2497//8174, rabbit Ab, 82‐97 kDa, Cell Signaling; 1:250//1:250); Anti‐GAPDH (ZG003, mouse monoclonal Ab, 37 kDa, Thermo Fisher, 1:500); Anti‐NOXA (14766, rabbit Ab, 10 kDa, Cell Signaling; 1:100); Anti‐human p21^Waf1/Cip1^ (P1484, mouse monoclonal Ab, 21 kDa, Sigma‐Aldrich, 1:250); Anti‐Phospho‐p53 (9284, rabbit Ab, 53 kDa, Cell Signaling, 1:500); Anti‐Rictor (9476, Cell Signaling, 200 kDa, 1:1000) and monoclonal Anti‐Na^+^/K^+^‐ATPase (clone M7‐PB‐E9, Sigma‐Aldrich, 110 kDa, 1:150).

#### Secondary antibodies

Stabilized peroxidase‐conjugated goat anti‐mouse (Thermo Scientific 1:5000) and Goat Anti‐Rabbit (Thermo Scientific; 1:5000). All antibodies were prepared in NaAzid free blocking buffer. Luminol and enhancer for western blotting were from Thermo Scientific.

### Inorganic solutions

Phosphate‐buffered saline (PBS) contained 137 mmol L^−1^ NaCl, 2.6 mmol L^−1^ KCl, 6.5 mmol L^−1^ Na_2_HPO_4_, and 1.5 mmol L^−1^ KH_2_PO_4_. Isotonic NaCl solution (320 mOsM) contained 152.5 mmol L^−1^ NaCl, 5 mmol L^−1^ KCl, 1 mmol L^−1^ Na_2_HPO_4_, 1 mmol L^−1^ CaCl_2_, 1 mmol L^−1^ MgSO_4_, plus 10 mmol L^−1^ N‐2‐hydroxyethyl piperazine‐N’‐2‐ethanesulfonic acid (HEPES). Hypotonic NaCl solution was prepared by reducing the NaCl concentration without changing the concentration of the other components. pH was in all solutions adjusted to 7.4 and osmolarities measured on a Knauer Osmometer Automatic.

### Cell cultures

Wild type, human lung adenocarcinomic alveolar epithelial cells (A549WT), were purchased from American Type Culture Collection (ATCC, Manassas, VA) and grown in Greiner Bio‐one tissue culture flasks (T75, 75 cm^2^ growth area) in DMEM supplemented with 10% fetal bovine serum (v/v) and penicillin / streptomycin (100 units penicillin/100 *μ*g streptomycin pr. ml). Cisplatin‐resistant A549 cells (A549RES) were developed by exposing A549WT cells to an increasing concentration (up to 10 *μ*mol L^−1^) of cisplatin for a period of 6 months and maintaining its resistant phenotype by treating the cells with 10 *μ*mol L^−1^ cisplatin every second passage (see (Sørensen et al. 2016b)). Wild‐type human embryonic kidney (HEK‐293) cells and HEK‐293 *lrrc8A* ^*−/−*^ (clone E7, gift from Prof. Thomas J. Jentsch FMP (Leibniz‐Institut fuer Molekulare Pharmakologie) and MDC (Max‐Delbrueck‐Centrum fuer Molekulare Medizin), D‐13125 Berlin, Germany) were cultured in 75 cm^2^ culture flasks (CellStar, Grenier Bio, Germany) in RPMI or DMEM media, respectively. Hypotonic growth media were obtained by dilution of the growth media with sterile water. All cell cultures were subcultured two times a week using 0.25% trypsine/EDTA in PBS and kept at 37°C, 5% CO_2_, and 100% humidity.

### Rictor knockdown

To eliminate mTORC2 activity transient knockdown of Rictor was carried out using a pool of four siRNA constructs against Rictor mRNA (SMARTpool: ON‐TARGETplus, GE Healthcare, Dharmacon). MISSION Universal Negative Control siRNA (Sigma Aldrich) was used to create a baseline for knockdown efficiency. Knockdown efficiency was estimated by western blot analysis of Rictor. Cells, grown to 50% confluence, were transfected with Rictor siRNA or negative control (scramble) siRNA at a concentration of 50 nmol/L using Lipofectamine 2000 Transfection Reagent (Thermo Fisher Scientific). After 4 h incubation, the medium was replaced by transfection reagent‐free medium, and cells incubated for another 20 h before analysis.

### SDS‐PAGE and western blotting

SDS‐PAGE and western blotting were used to quantify changes in protein levels. Cells were grown to 80‐90% confluence in a 6 cm petri dish, washed in ice cold PBS cells, and lysed in lysis buffer containing 1% Sodium dodecyl sulfate (SDS), 150 mmol L^−1^ NaCl, 20 mmol L^−1^ HEPES, 1 mmol L^−1^ EDTA, 0,5% Triton X‐100, 1 mmol L^−1^ Na_3_VO_4_ and 1% protease inhibitor cocktail. The lysates were sonicated before centrifugation (20,000 *g*, 5 min, 5°C) to precipitate insoluble cell material. The protein content in the supernatant was determined using a Bio‐Rad DC Protein Assay (Bio‐Rad, Hercules, CA) and lysates subsequently diluted in ddH_2_O to 20–40 *μ*g per loading. Protein samples were mixed with NuPAGE sample buffer containing dithiothreitol (DTT) and proceeded for SDS‐PAGE gel electrophoresis (NuPAGE precast 10% or 4–12% Bis‐tris gels in NuPAGE MOPS SDS running buffer, Invitrogen, Waltham, MA) in NOVEX chambers under reducing/denaturing conditions. To indicate the molecular weight of proteins we used a benchmark protein ladder (Invitrogen). Following protein separation by electrophoresis, we used NuPAGE transfer buffer (Invitrogen) for protein transfer to nitrocellulose membranes (Frisinette, Denmark). Successful protein transfer was verified by Ponceau S staining. Unspecific protein‐binding to membranes was prevented by incubation of the membranes in TBST (0.01 mol/L Tris–HCl, 0.15 mol/L NaCl, 0.1% Tween 20, pH 7.4) containing 5% nonfat dry milk at 37°C for 1 h. Membranes were incubated with primary antibodies diluted in blocking buffer overnight at 4°C before being washed in TBST and exposed to secondary antibodies for 1 h at room temperature. Membranes were developed using BCIP/NBT (KPL, Gaithersburg, MD), scanned and bands quantified using UN‐SCAN‐IT (Silk Scientific).

### Determination of rate constants for taurine release and inactivation of the VRAC complex

Swelling‐induced taurine efflux was estimated at room temperature in A549 and HEK cells grown to 80% confluence in six‐well polyethylene culture (cells) or Poly‐Lysine coated (Greiner Bio‐One) plates, respectively, as previously described (Sørensen et al. 2016b). Each well represented one release experiment. Cells were loaded with [^3^H]taurine (37000 Bq/well) in growth medium for 2 h (37°C, 5% CO_2_, 100% humidity). Taurine release experiments were initiated by 3 times wash of the wells with 1 mL isotonic NaCl medium to remove residual extracellular isotope and growth media. Release of [^3^H]taurine was subsequently followed with time by transferring the NaCl medium from each well to individual scintillation vials (Snaptwist Scintillation vial, 6.5 mL, VWR) and replacing it with fresh medium at 2‐min (A549 cells) or 5‐min (HEK cells) intervals. The experiment was run for the total of 30–40 min with the isotonic medium being replaced by hypotonic media 10 min after initiation of the efflux experiment. At the end of the assay, remaining intracellular [^3^H]taurine was extracted by addition of 1 mL 1 mol/L NaOH (1 h, shaking table) followed by two times wash with 1 mL ddH_2_O. 3.5 mL scintillation fluid was added to each vial and [^3^H]‐activity detected by *β*‐scintillation counting (Perkin Elmer scintillation counter, Waltham, MA). Total [^3^H]activity in the cell system was calculated as the sum of [^3^H]activity released during the efflux experiment and in the NaOH/water washouts. The fractional rate constant for taurine release (k, min^−1^) was calculated from the equation: k = [ln(X_1_) − ln(X_2_)]/(*t*_1_–*t*_2_) (X_1_ and X_2_ denote the fraction remaining in the cell at time *t*_1_ and *t*_2_, respectively) and plotted versus time. The rate constant for taurine release under isotonic conditions was determined as the mean of the 2–3 values obtained prior to the hypotonic exposure. Maximal swelling‐induced rate constant was determined as the highest rate constant following hypotonic exposure. Inactivation of the VRAC complex activity at a given time point “*t*” following hypotonic activation of its activity was determined as (k^max^–k^*t*^)/(k^max^–k^iso^), where k^max^, k^iso^ are the maximal and the isotonic rate constant and k^t^ the constant at time “*t*”.

### Quantitative real‐time polymerase chain reaction (qRT‐PCR)

qRT‐PCR was performed to quantify LRRC8A, LRRC8D, and Actin‐related protein 2/3 ARP2/3 (house‐keeping gene) mRNA accumulation as previously described (Sørensen et al. 2014). Nucleospin^®^ RNA II kit (MACHEREY NAGEL, Germany) was used for RNA isolation from cells grown to 90% confluence in 10 cm petri dishes. Reverse transcription was performed by incubating 1 *μ*g RNA (NanoDrop; Thermo Fisher Scientific) with nucleotide solution (dNTP mix) plus oligo dT primer at 65°C for 5 min and subsequently with strand buffer plus dithiothreitol (DTT) at 42°C for 2 min. Superscript II reverse transcriptase was added and mixture incubated at 42°C for 50 min. The transcription reaction was stopped by raising the temperature to 70°C for 15 min. mRNA accumulation was determined with Brilliant II SYBR^®^ Green QPCR master mix plus predesigned and validated KiCqStart^®^ SYBR^®^ Green Primers (Sigma Aldrich), for example: LRRC8A (Fwd: GGGTTGAACCATGATTCCGGTGAC//Rev: GAAGACGGCAATCATCAGCATGAC; LRRC8D (Fwd: ATGGAGGAGTGAAGTCTCCTGTCG//Rev: CTTCCGCAAGGGTAAACATTCCTG); ARP (Fwd: CGACCTGGAAGTCCAACTAC //Rev: ATCTGCTGCATCTGCTTG). qRT‐PCR was performed using the following conditions: 10 min at 95°C followed by 40 cycles (30 sec at 95°C, 1 min at 60°C, 1 min at 72°C) and a single final elongation step for 3 min at 72°C. Mean *C*_T_ value was calculated and LRRC8A/LRRC8D were normalized to ARP2/3.

### LRRC8A mutants

The LRRC8A‐GFP expression vector was generated previously (Sørensen et al. 2016b). The LRRC8A mutants were made by homologous recombination. Yeast cells were transformed with BamHI. SalI and HindIII (Fermentas, Thermo Fischer Scientific) digested pPAP7160 vector (Sørensen et al. 2016b) and two LRRC8A cDNA fragments, an upstream and a downstream cDNA fragment both containing the mutation sites. The cDNA fragments were obtained through regular PCR using AccuPol polymerase (VWR, Denmark), a human LRRC8A‐EGFP cDNA ORF clone (Sørensen et al. 2016b) and the following primers: LRRC8A (forward): 5′‐ATA TAA GCA GAG CTG GTT TAG TGA ACC GTC AGA TCG GGT TGA ACC ATG ATT CCG GTG ACA GAG CT‐3′; LRRC8A (reverse): 5′‐ACC CCG GTG AAC AGC TCC TCG CCC TTG CTC ACC ATG GCC TGC TCC TTG TCA GC‐3′; LRRC8A(*p.Tyr382Phe)* (forward): 5`‐TCA TTG ACC AAT TTG ACC CGC TC‐3`; LRRC8A(*p.Tyr382Phe)* (reverse): 5`‐GAG CGG GTC AAA TTG GTC AAT GA‐3`; LRRC8A(*p.Tyr386Phe)* (forward): 5`‐GAC CCG CTC TTT TCC AAG CGC‐3`; LRRC8A(*p.Tyr386Phe)* (reverse): 5`‐ GCG CTT GGA AAA GAG CGG GTC ‐3`; LRRC8A(*p.Lys501Arg)* (forward): 5`‐GCT GCA CAT CAG GTT CAC CGA CA‐3`; LRRC8A(*p.Lys501Arg)* (reverse): 5`‐TGT CGG TGA ACC TGA TGT GCA GC‐3` (TAG Copenhagen A/S, Denmark). The 3`‐LRRC8A PCR product carried a 35‐nucleotide sequence identical to the 5′‐coding sequence of EGFP on the pPAP7160 vector, whereas the 5′‐LRRC8A PCR product carried a 35‐nucleotide sequence identical to the pPAP7160 vector upstream to the restriction enzyme digestion‐site (Sørensen et al. 2016b).

The yeast cells were grown for 2–3 days at 37°C on Leu‐Lys‐agar plates added 0.5% V‐200 and 8 ng/mL tetracycline. Following incubation, 2–3 colonies were collected and transferred to Leu‐Lys media. Leu‐Lys media contained 10% ASD‐10, 2% glucose, 0.01% CaCl_2_, 1% leucine, and 1% lysine in ddH2O (autoclaved before use) and added 0.5% V‐200 and 8 ng/mL tetracycline. The yeast cells were grown for additional 2 days on a shaker at 37°C. Uracil‐positive cells were used to select for the presence of a rejoined plasmids obtained by homologous end‐joining with the two cDNA fragments. Hereafter, the transformed yeast cells were lysed by centrifugation (3000 *g*, 5 min), addition of 1:1 lyticase buffer (100 mmol L^−1^ Tris‐HCl, pH 8.0, 100 mmol L^−1^ EDTA and 2/3 LB) and 10 *μ*L lyticase (5 unit/*μ*L in TE buffer, Sigma). The yeast cells were incubated 1 h at 37°C and shaken thoroughly. Afterwards, the cells were transferred to 10 *μ*L 20% SDS, mixed, and frozen (−80°C). The LRRC8A‐expression vector was purified using a NucleoSpin Plasmid DNA purification kit (MACHEREY NAGEL). DNA yield was amplified by E. coli transformation (OmniMax cells), followed by NucleoBond Xtra Midi Plasmid DNA Purification (MACHEREY NAGEL). The correct nucleotide sequences were confirmed by DNA sequencing (Eurofins Genomics, Germany). The plasmid concentrations were measured on NanoDrop (Thermo Fischer Scientific).

### Plasmid transfection of HEK‐293 lrrc8a^−/−^ cells

HEK‐293 *lrrc8a* ^−*/*−^ cells grown to 70% confluence in six‐well plates were transfected with empty vector (pPAP7160), LRRC8A‐EGFP, or one of the three mutants (LRRC8A‐p.Tyr382Phe‐EGFP, LRRC8A‐p.Tyr386Phe‐EGFP or LRRC8A‐p.Lys501Arg‐EGFP). The cells were transfected with 2500 ng plasmid/well (total volume = 2 mL DMEM/well) using Lipofectamine™ 2000 Transfection Reagent (5 *μ*L per transfection). Briefly, Lipofectamine™ and the individual plasmids were each mixed with 100 *μ*L DMEM transfection media (i.e., without penicillin/streptomycin and FBS) and incubated for 5 min at RT. During incubation, the cell culture media covering the cells was replaced by 1.6 mL DMEM transfection media. Following incubation, the transfection reagent and the plasmid solutions were mixed together and left for another 30 min at room temperature. Finally, the transfection mixture was dripped over the cells and the cells were added 200 *μ*L FBS/well. The cells were incubated 24 h before initiation of the experiment in the cell culture incubator. For the taurine release experiments, the cell culture media was changes to transfection‐reagent‐free DMEM media containing FBS before loading the cells with [^3^H]taurine.

### Cell surface biotinylation and membrane protein isolation

Cells, grown to 80% confluence in four T75 flasks (each), were biotinylated and labeled proteins isolated according to the manufactures instructions in the Pierce Cell Surface Protein Isolation Kit (Thermo Fisher Scientific). SDS‐PAGE and western blotting were used to analyze the expression of LRRC8A and Na^+^/K^+^‐ATPase (positive plasma membrane control / loading control) in both whole cell lysate and purified samples.

### Statistics

All data are statistically tested (SigmaPlot version 12) by one‐way ANOVA with Fisher LSD Method as posttest or by Student′s *t*‐test. In bar‐ and scatter‐plots the error bars signify standard error of the mean (SEM).

## Results

### Effect of hypotonic adaptation on LRRC8A expression and volume‐sensitive taurine release

**Figure 1:**
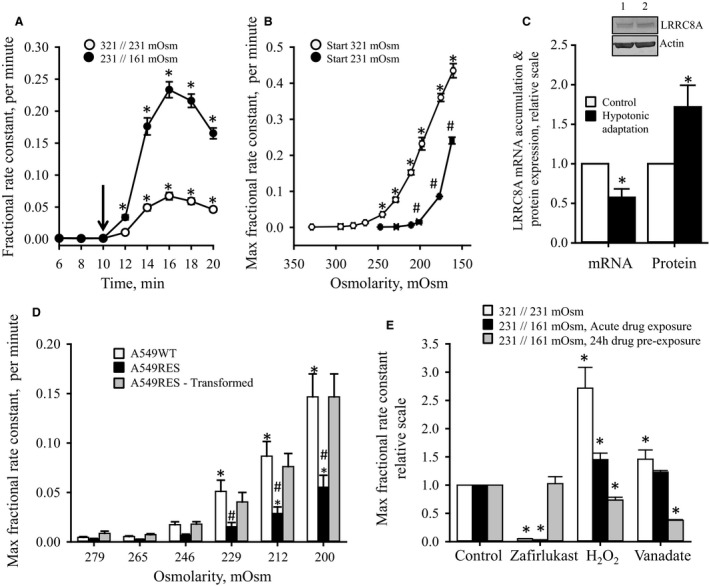
Swelling induced release of taurine and LRRC8A mRNA/protein expression in A549 cells following repetitive hypoosmotic exposure or exposure to varying degrees of hypoosmotic challenge. Taurine release, mRNA accumulation and protein expression, were measured by tracer technique, qRT‐PCR and SDS‐PAGE/western blotting, respectively. (A) Fractional rate constants (min^−1^) for [^3^H]taurine release from cisplatin‐sensitive A549 (A549WT) cells, preloaded with [^3^H]taurine, were determined under isotonic/hypotonic conditions and plotted versus time. One group of cells, preincubated in isotonic growth medium, was exposed for 10 min to isotonic Ringer (321 mOsm) before exposure to hypotonic Ringer (321//231 mOsm) (○, *n* = 10). Another group, adapted for 24 h in hypotonic growth medium (220–226 mOsm), was exposed for 10 min to hypotonic Ringer with a similar tonicity (231 mOsm) followed by an additional hypotonic challenge (231//161 mOsm Ringer) (●, *n* = 9). Shift in tonicity is indicated by the arrow. Data represent mean values ± SEM. *indicates statistical increased compared to the prehypotonic values (*P* < 0.05, ANOVA, Fisher LSD Method). (B) Set‐point for volume‐sensitive taurine release in A549WT cells, preexposed to isotonic medium (○, *n* = 4) or hypotonic medium (●, *n* = 3), before exposure to Ringers within the range 329–161 mOsm (○) or 250–161 mOsm (●), respectively. Fractional rate constants for taurine release were at each tonicity followed as function of time, as indicated in A, and the maximal fractional rate constant determined as the rate constant 6 min following shift in tonicity. *indicates statistically increased compared to the rate constant before hypotonic challenge, that is, 0.0010 ± 0.00004 min^−1^ (○, 321 mOsm) and 0.0015 ± 0.00006 min^−1^ (●, 231 mOsm) (*P* < 0.05, ANOVA, Fisher LSD Method). (C) LRRC8A mRNA accumulation and protein expression were determined in A549WT cells grown in isotonic growth medium (control, open bars) or adapted for 24 h to hypotonic growth medium (220–226 mOsm, black bars). ***Insert:*** Representative western blots for LRRC8A and actin (*Lane 1*: Control cells; *Lanes 2*: Hypotonically adapted cells). Values for hypotonic adapted cells are given relative to control cells and represent means ± SEM of 4 (mRNA) and 6 (protein) sets of independent experiments. *indicates statistical different compared to control cells (*P* < 0.05, paired Student′s *t*‐Test). (D) Volume set point for activation of the volume‐sensitive taurine release was determined in A549WT (open bars) and cisplatin‐resistant A549 cells (A549RES) (black bars) as the maximal rate constant obtained 6 min after exposure to hypotonic media in the range 279–200 mOsm. Maximal rate constant are given as mean values ± SEM from 3 sets of independent experiments. The aligned values (gray bars) for A549RES were obtained by multiplication of A549RES values with the ratio between the maximal rate constant for A549WT and A549RES after exposure to hypotonic medium (200 mOsm). *indicates statistical increased compared to rate constants obtained under isotonic conditions (*P* < 0.05, ANOVA, Fisher LSD Method). (E) Effect of H_2_O_2_, vanadate and zafirlukast preexposure on the maximal fractional rate constant for taurine release following hypoosmotic challenge. Rate constants for swelling‐induced release of [^3^H]taurine were plotted as function of time (see Panel A) for A549WT cells preexposed to 321 mOsm medium before hypoosmotic challenge in 231 mOsm (open bars) or cells adapted to 231 mOsm for 24 h (black and gray bars) before hypoosmotic challenge in 161 mOsm, respectively. Maximal rate constants, obtained 6 min after hypoosmotic exposure, were determined in the absence (control, open bars), following acute exposure (black bars) or 24 h pre‐exposure (gray bars) to H_2_O_2_ (100 *μ*mol L^−1^), vanadate (50 *μ*mol L^−1^) or Zafirlukast (60 *μ*mol L^−1^). Maximal fractional rate constants, are given relative to the maximal constant for the respective control cells, that is, 0.067 ± 0.006 min^−1^ (321//231 mOsm; *n* = 10) and 0.234 ± 0.012 min^−1^ (231//161 mOsm; *n* = 10). *indicates statistical different compared to control values (*P* < 0.05, ANOVA, Fisher LSD Method).

Figure 1A shows, in congruence with previously published data (Holm et al. 2013), that release of taurine from A549 is transiently increased in response to hypotonic exposure**.** Cells, grown in standard DMEM medium, were subjected to a 28% decrease in osmolarity (321☐ 231 mOsm), whereas cells adapted for 24 h to hypotonic growth medium (220–226 mOsm for 24 h) were subjected to a 30% decrease in osmolarity (231▫161 mOsm). In both cases, cells obtained a maximal fractional rate constant for taurine release within 6 min following hypotonic exposure, that is, 0.06 min^−1^ and 0.23 min^−1^ for control cells and hypotonically adapted cells, respectively. Inactivation of the volume‐sensitive taurine release pathway is almost complete after 24 h exposure to the 231 mOsm medium, that is, the rate constant for cells adapted to 231 mOsm medium was 0.0015 ± 0.00006 min^−1^ (*n* = 3) compared to 0.0010 ± 0.00004 min^−1^ (*n* = 8) for control cells in isotonic 321 mOsm. To investigate the difference in the maximal fractional rate constants, following a quantitatively similar osmotic challenge, that is, 28–30% reduction in tonicity, we tested how control cells and hypotonically adapted cells responded to increasingly larger osmotic challenges. From Figure 1B it is seen that the maximal rate constants for the swelling‐induced taurine release for cells, incubated in 321 mOsm medium, is significantly activated after a 25% reduction in osmolarity (321→245 mOsm; fractional rate constant = 0.035 min^−1^). However, the hypotonically adapted A549 cells revealed a significant activation after 13% decrease in tonicity (231→201 mOsm; fractional rate constant = 0.015 min^−1^). Furthermore, after the “set‐point,” that is, the cell volume required for activation of the VRAC complex, has been reached, the relationship between medium osmolarity and the maximal rate constant for the swelling‐induced taurine release, in control cells and hypotonically adapted cells, increases in what appears to be a sharp escalation (Fig. 1B). Similar correlation between activation of volume‐sensitive taurine efflux and anisotonic solutions has previously been demonstrated for bovine articulate chondrocytes (Hall and Bush 2001). The difference in maximal fractional rate constants observed for A549 cells (Fig. 1A) thus reflects that the shift in hypotonically adapted cells from 231 mOsm to 161 mOsm ends in the steeper part of the curve, whereas shift from 321 mOsm to 231 mOsm in control cells barely reaches the steep part of the escalation curve (Fig. 1B). From Figure 1C it is seen that although hypotonic adaptation reduces LRRC8A mRNA accumulation by approximately 40%, the LRRC8A protein expression is concomitantly increased 1.6 fold. Hence, higher LRRC8A protein expression following hypotonic adaptation does not reflect an increase LRRC8A gene transcription. Whether, the reduced LRRC8A mRNA accumulation reflects a putative negative feedback from an increased LRRC8A protein expression was not investigated.

To test whether an increased LRRC8A expression could affect the set‐point for the volume‐sensitive taurine release in A549 cells we exposed cisplatin‐sensitive (A549WT) and cisplatin‐resistant A549 (A549RES) cells to hypotonic challenges in the range 279–200 mOsm. The A549RES cells were included, because we have previously shown that although total LRRC8A protein expression is significantly increased in the A549RES cells the VRAC complex activity is low due to a reduced LRRC8A availability in the plasma membrane (Sørensen et al. 2016b). Figure 1D shows, in congruence with our previous data (Sørensen et al. 2016b), that the swelling‐induced taurine release is reduced in A549RES cells compared to the A549WT cells following exposure to hypotonic media (321 mOsm→279..200 mOsm). To align the osmosensitivity in the two cell lines we multiplied the maximal rate constant for the swelling‐induced taurine release in A549RES cells by the ratio between maximal rate constants for A549WT and A549RES after exposure to 200 mOsm (Lambert 2003). From Figure 1D we see that the transformed values for taurine release in A549RES and A549WT cells are identical within the osmolarity range tested (compare open and gray bars). Hence, a reduced LRRC8A availability in the plasma membrane, as seen in A549RES, does not shift the set‐point for activation of the VRAC complex.

To test whether prevention or boosting of taurine loss during a hypotonic exposure would affect taurine loss during a subsequent hypotonic exposure we exposed cells to the CysLT1 receptor antagonist zafirlukast or to H_2_O_2_. In agreement with earlier observations in A549 cells (Holm et al. 2013), acute exposure to the zafirlukast, elicits a 95% reduction in the swelling‐induced taurine release in control and hypotonically adapted cells (Fig. 1E). However, zafirlukast mediated prevention of taurine loss during the first hypotonic challenge, has no effect on the subsequent secondary swelling‐induced taurine release performed after zafirlukast washout (Fig. 1E). It has previously been shown that the ROS production increases following hypotonic exposure and that ROS boost volume‐sensitive taurine release (Lambert 2003; Friis et al. 2008; Lambert et al. 2009; Holm et al. 2013). However, Figure 1E shows that whereas acute exposure to H_2_O_2_ potentiates the swelling‐induced taurine release 2.71 fold in control cells exposed to hypotonic medium (321→231 mOsm), this effect is reduced in hypotonically preexposed cells (231→161 mOsm) and even reversed into an inhibition in cells preexposed for 24 h in hypotonic medium containing H_2_O_2_. This could indicate that ROS, generated under the first hypotonic stress, limit osmolyte loss under a subsequent hypotonic challenge and that the effect depends on the ROS concentration. From Figure 1E it is also seen that the effect of H_2_O_2_ preexposure on swelling‐induced taurine release is mimicked by the tyrosine phosphatase inhibitor vanadate. Cysteines are sensitive to oxidation and as they are found in the active site of protein tyrosine phosphatases (PTP) it is assumed that the effect of ROS partly reflects oxidative inhibition of PTP and consequently enhancement of the effect of opposing protein tyrosine kinases (Lambert et al. 2015b). In this context it is noted that volume‐sensitive taurine release in A549 has been shown to be inhibited by the general tyrosine kinase inhibitor Genistein and the selective Janus kinase (JAK) inhibitor cucurbitacin (Holm et al. 2013). Whether, down‐regulation of the ROS sensitivity of the volume‐sensitive taurine release following adaptation to hypotonic conditions and/or chronic ROS exposure reflects reduced tyrosine kinase expression/activity was not investigated further.

### Effect of oxidative stress (H_2_O_2_) on LRRC8A subunit expression, VRAC activity, and apoptotic cell signaling

**Figure 2:**
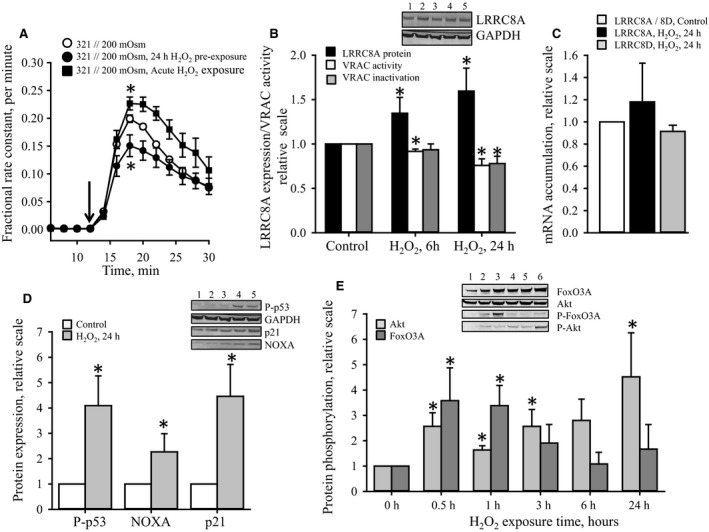
Effect of exogenous H_2_O_2_ on LRRC8A mRNA accumulation, LRRC8A protein expression, swelling induced taurine release, and intracellular signaling associated with apoptosis and cell growth. Taurine release, mRNA accumulation and protein expression in A549WT were measured by tracer technique, qRT‐PCR and SDS‐PAGE/western blotting, respectively. (A) Fractional rate constant (min^−1^) for [^3^H]taurine release was determined in A549WT cells under isotonic conditions (321 mOsm) and following reduction in the osmolarity to 200 mOsm (shift in tonicity indicated by the arrow) in the absence (control, open circles) or presence of 100 *μ*mol L^−1^ H_2_O_2_ (acute exposure, closed squares). Alternatively, cells were preexposed to isotonic growth medium containing 100 *μ*mol L^−1^ H_2_O_2_ for 24 h prior to the release experiment (closed circles). Rate constants at each time point represent mean values from 7 (Control), 4 (preexposed) and 4 (acute exposure) sets of experiments. *indicates that maximal rate constant, obtained 6 min after hypotonic exposure, was significantly different from control cells (*P* < 0.05, paired Student′s *t*‐Test). (B) A547WT cells were preexposed to isotonic growth medium containing 100 *μ*mol L^−1^ H_2_O_2_ for 6 or 24 h prior to taurine release experiments for determination of maximal rate constants (min^−1^), inactivation of the VRAC complex activity and LRRC8A protein expression. Values ± SEM for maximal rate constant, inactivation of taurine release pathway (4 sets with 6 and 24 h exposure) and protein expression (4/10 sets for 6/24 h exposure, respectively) are given relative to control cells not exposed to H_2_O_2_. ***Insert:*** Representative western blots for LRRC8A and GAPDH (*Lane 1*: Control cells; *Lanes 2 and 3*: Cells exposed to 25 *μ*mol L^−1^ H_2_O_2_ for 6 and 24 h, respectively; *Lanes 4 and 5*: Cells exposed to 100 *μ*mol L^−1^ H_2_O_2_ for 6 and 24 h, respectively). *indicates that values were significantly different from the control cells (*P* < 0.05, paired Student′s *t*‐Test). (C) LRRC8A and LRRC8D mRNA accumulation was determined in control A549WT cells and cells exposed for 24 h to 100 *μ*mol L^−1^ H_2_O_2_. Values, calculated relative to untreated control cells, are given as means ± SEM of 4 sets of experiments. (D) A549WT cells were incubated in growth medium containing 100 *μ*mol L^−1^ H_2_O_2_ for 24 h before proteins were extracted and tested for expression of phosphorylated p53 (P‐p53), NOXA, p21, and GAPDH (house holding protein). Protein expression was normalized to GAPDH (9, 5, and 8 sets for P‐p53, NOXA and p21, respectively), and presented as means ± SEM of values relative to non‐treated control cells. ***Insert:*** Representative western blots for P‐p53, p21, NOXA and GAPDH for (*Lane 1*: Control cells; *Lanes 2 and 3*: Cells exposed to 25 *μ*mol L^−1^ H_2_O_2_ for 6 and 24 h, respectively; *Lanes 4 and 5*: Cells exposed to 100 *μ*mol L^−1^ H_2_O_2_ for 6 and 24 h, respectively). *indicates that the protein expression of apoptotic markers was significantly different from control cells (*P* < 0.05, paired Student′s *t*‐Test). (E) A549WT cells were incubated in growth medium containing 100 *μ*mol L^−1^ H_2_O_2_ for 24 h. Proteins were extracted at the time points indicated and tested for expression of total and phosphorylated Akt / FoxO3A. Protein expression ratios between phosphorylated and total proteins were calculated in 4 sets of experiments and data presented as mean values ± SEM of the ratios relative to control cells. ***Insert:*** Representative western blots for FosO3A, Akt, P‐FosO3A, and P‐Akt (*Lane 1*: Control cells; *Lanes 2 to 6*: Cells exposed to 100 *μ*mol L^−1^ H_2_O_2_ for ½, 1, 3, 6, and 24 h, respectively). *indicates that the protein expression was significantly different from control cells (*P* < 0.05, paired Student′s *t*‐Test).

In accordance with the data in Figure 1 the time traces in Figure 2A show that following hypotonic exposure (321→200 mOsm) the swelling‐induced taurine release is boosted by H_2_O_2_, added acutely, but inhibited when H_2_O_2_ is present for 24 h prior to the subsequent hypotonic challenge (maximal rate constants: Control 0.20 ± 0.007 min^−1^; Acute ROS exposure 0.23 ± 0.02 min^−1^; ROS preexposure 0.15 ± 0.02 min^−1^). However, Figure 2B illustrates an inverse correlation between LRRC8A protein expression and the volume‐sensitive taurine release following preexposure to H_2_O_2_, that is, protein expression is increased 1.3 and 1.5 fold following 6 and 24 h of exposure to H_2_O_2_, respectively, whereas the maximal fractional release constant is concomitantly reduced by 8% and 24% compared to control cells. Furthermore, ROS preexposure also reduces the relative inactivation of the taurine release pathway (Fig. 2B), that is, 20 min posthypotonic exposure the inactivation was 62 ± 2%, 59 ± 5% and 49 ± 6% for control and H_2_O_2_ preexposed for 6 h or 24 h cells, respectively (100% inactivation would have indicated that the rate constant had reached the isotonic value). It is noted that a reduced inactivation, that is, an increased open probability of the VRAC complex could compensate for a reduced maximal activity in relation to net loss of osmolytes. However, at the end of the release experiment (time 30 min) the control cells and cells preexposed to H_2_O_2_ contained 10 ± 1% (*n* = 7) and 17 ± 5% (*n* = 4) of their original pool of [^3^H]taurine, respectively, that is, prolonged exposure to H_2_O_2_ reduces volume‐sensitive net loss of taurine in A549 cells. Hence, although H_2_O_2_ preexposure increases LRRC8A protein expression this does not govern a higher volume‐sensitive taurine release in A549 cells. From Figure 2C it is seen that the mRNA accumulation for LRRC8A and LRRC8D, which together constitutes the volume‐sensitive taurine release pathway, is unaffected by 24 h incubation with H_2_O_2_. These data indicate that upregulation in LRRC8A protein expression, following cell stress (hypotonic or H_2_O_2_ exposure), does not imply shift in the LRRC8A gene transcription.

Cell stress is known to instigate apoptosis and/or cell cycle arrest. From Figure 2D it is seen that H_2_O_2_ exposure causes a 4.1, 2.3, and 5.0 fold increase in the expression of phosphorylated p53 (transcription factor indicating activation of proapoptotic transcription), NOXA (Bcl‐2 family member indicating progression of apoptosis) and p21 (cyclin‐dependent kinase inhibitor p21 indicating cell growth inhibition), respectively. This indicates that an increase in LRRC8A expression, following prolonged H_2_O_2_ exposure_,_ correlates with instigation of apoptosis and inhibition of cell cycle progression. The Forkhead box proteins FoxO1, FoxO3 and FoxO4 are ubiquitously expressed transcription factors that similar to p53 act as tumor suppressors though promotion of cell cycle arrest and instigation of apoptosis (Morris et al. 2015). FoxOs are negatively regulated through PI3K‐Akt signaling, that is, FoxO3 mediated transcription is prevented by Akt mediated phosphorylation (T32, S243, S315) and nuclear export. However, FoxO3 phosphorylation by the AMP‐dependent kinase AMPK (S413) does not shift its localization and instead leads to an increased transcriptional activity (Greer et al. 2007). From Figure 2E (western blot insertion) it is seen that the expression of FoxO3 is dramatically increased within hours following H_2_O_2_ exposure, which is taken to indicate that the H_2_O_2_ induced increase in LRRC8A expression, correlates progression of apoptosis through p53 and presumably FoxO3 mediated activity. However, although phosphorylation of FoxO3 on S413 initially is significantly increased by H_2_O_2_ it diminishes with time concomitantly to the increase in Akt activity, indicated as the ratio between the expression of phosphorylated Akt and total Akt (Fig. 2E).

### Effect of cisplatin exposure on LRRC8A subunit expression, VRAC activity, and growth‐associated signaling

**Figure 3:**
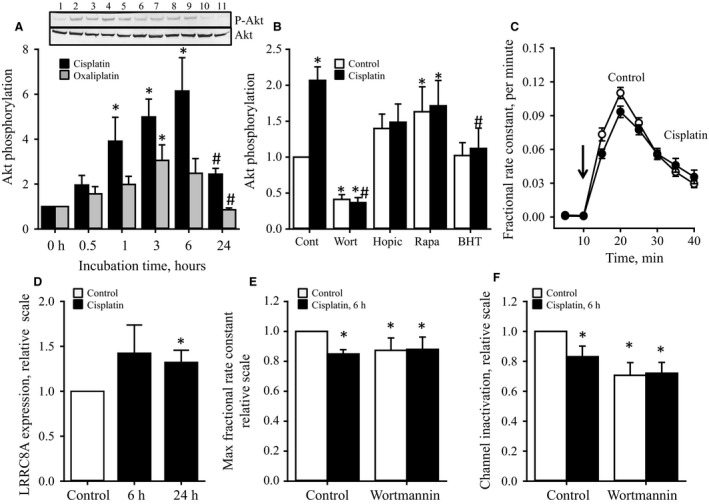
Effect of cisplatin on Akt phosphorylation, swelling‐induced taurine release, and VRAC inactivation. Protein expression was determined by SDS‐PAGE/western blotting (WB) followed by protein quantification using UN‐SCAN‐IT. Rate constants and inactivation for the volume‐sensitive taurine release were obtained by tracer technique. (A) Transient Akt activation in A549 cells following exposure to cisplatin and oxaliplatin. Cells were exposed to 20 *μ*mol L^−1^ cisplatin or oxaliplatin. Lysates were taken and tested for P‐Akt and total Akt expression at the time points indicated. P‐Akt/Akt expression ratios from 3 sets of experiments were in each setup calculated relative to the untreated controls and presented as mean values ± SEM. *Insert:* Representative P‐akt and total Akt western blots (*Lane 1*: Control cells; Lanes 2–6: 0.5 , 1, 3, 6, 24 h exposure to cisplatin; Lanes 7–11: 0.5, 1, 3, 6, 24 h exposure to 20 *μ*mol L^−1^ oxaliplatin). * and # denote that P‐Akt / Akt expression ratios are statistically different from the ratio in untreated control or the maximal P‐Akt/Akt ratio (time 6 h), respectively (one‐way ANOVA with Fischer's LSD test. (B) A549 cells were exposed for 6 h to Wortmannin (Wort, 5 *μ*mol L^−1^), HOpic (5 *μ*mol L^−1^), Rapamycin (Rapa, 400 nmol/L) or BHT (0.5 mmol L^−1^) in the absence or presence of 20 *μ*mol L^−1^ cisplatin. Lysates were taken and proceeded for detection of total‐Akt and P‐Akt. Values indicate P‐Akt/Akt expression ratios from 4 sets of experiments were in each setup calculated relative to the untreated controls and presented as mean values ± SEM. * and # denote statistically different from cells not treated with cisplatin and cells treated with cisplatin, respectively (*P* < 0.05, one‐way ANOVA with Fischer's LSD test). (C, E, F) A549 cells were exposed to 20 *μ*mol L^−1^ cisplatin for 6 h before protein extraction/western blotting or initiation taurine release experiment. Fractional rate constant (min^−1^) for taurine release was determined from release of [^3^H]taurine and plotted versus time under isotonic and hypotonic conditions (C, shift in tonicity indicated by the arrows). Maximal hypotonic taurine release (E) were obtained from the time traces and set relative to control cells not exposed to cisplatin. The VSOAC inactivation (F) was calculated as indicated in Materials and Methods. The data represent mean values from five sets of experiments ± SEM. *Statistically different from untreated control (Student *t*‐test). (D) LRRC8A protein expression was determined in control and cells exposed for 6 h to cisplatin. Values are given relative to untreated control cells and represent mean ± SEM of 3 sets of experiments. *Statistically different from untreated control (*P* < 0.05, Student *t*‐test).

Activation of the serine/threonine kinase Akt, which is normally associated with cell growth, has in several studies been associated with modulation of volume‐sensitive taurine release (Lambert et al. 2015b) as well as development of cisplatin resistance (Gagnon et al. 2004; Winograd‐Katz and Levitzki 2006; Hahne et al. 2012). In human breast (MDA MB‐468) carcinoma cells, it has been shown that cisplatin activates Akt in a way that depends on the epidermal growth factor receptor (EGFR) and Src kinase (Winograd‐Katz and Levitzki 2006). Moreover, in human lung A549 cancer cells Akt‐1 amplification was found to promote cisplatin resistance through the mTORC1/p70S6K‐1 signaling pathway (Liu et al. 2007). We have investigated the effect of cisplatin and oxaliplatin on the activation of Akt, measured as its Ser‐473 phosphorylation. Figure 3A, representative western blot (insert) and quantification, shows that cisplatin and oxaliplatin induce a transient activation of Akt within 24 h where maximal activity is obtained following 6 h treatment. The Akt activation is inhibited by co‐treatment with the PI3K inhibitor Wortmannin, indicating that the cisplatin‐induced Akt activation depends on the PI3 kinase (Fig. 3B). Moreover, whereas the PTEN inhibitor HOpic and the mTORC1 inhibitor rapamycin slightly potentiate phosphorylation of Akt in the absence of cisplatin (Fig. 3B), they have no significant effect on the cisplatin‐induced Akt activation (Fig. 3B). However, co‐treatment with the anti‐oxidant BHT significantly reduces the cisplatin‐induced Akt activity, indicating that activation of cisplatin induced Akt activation involves ROS (Fig. 3B).

**Figure 4:**
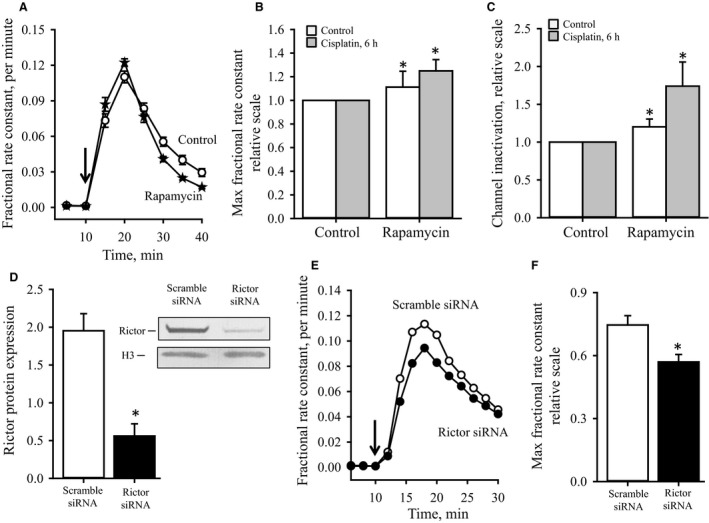
Role of mTORC1 and mTORC2 in regulation of swelling‐induced taurine release. Cells were pretreated with cisplatin in the absence or presence of rapamycin or siRNA. (A) Time course for taurine release under isotonic and hypotonic conditions in control cells or cells preexposed to rapamycin (400 nmol L^−1^, 6 h). Values are mean of 3–6 sets of experiments. (B and C) Maximal hypotonic taurine release (B) and inactivation of the release pathway (C) were obtained as indicated in the legend to Figure 3 from the time traces in Panel A and presented as mean values of 3–6 sets of experiments ± SEM. *Statistically different from untreated control cells (*P* < 0.05, Students *t*‐test). (D–F) A549 cells were transfected with scramble or Rictor siRNA for 48 h. (D) Rictor expression relative to histone (H3, house holding protein) expression was determined from four sets of paired experiments and presented as mean values ± SEM. *Insert*: Representative Richtor and H3 western blots. *Statistically different from untreated control (Student *t*‐test). (E) Fractional rate constant (min^−1^) for taurine release versus time under isotonic and hypotonic conditions (shift in tonicity is indicated by the arrow) in scramble (open circles) and Rictor (closed circles) siRNA transfected A549 cells (one representative experiment is shown). (F) Maximal hypotonic taurine release constants were obtained from the time traces and set relative to the scramble siRNA transfected controls. Data in C represent mean of 3 (scramble siRNA) and 6 (Rictor siRNA) sets of experiments ± SEM. *Statistically different from control (*P* < 0.05, Student *t*‐test).

Short‐term exposure to cisplatin has previously been demonstrated to increase LRRC8A protein in human ovarian carcinoma (A2780) cells significantly (Sørensen et al. 2014). From the data presented in Figure 3C–F it is seen that although 6–24 h pretreatment with cisplatin increases the expression of LRRC8A protein, it reduces the swelling‐induced taurine release and delays channel inactivation. Figures 3E and F also show that inhibition of PI3K activity by addition of wortmannin not only limits the swelling‐induced taurine release and delays inactivation of the VRAC complex but also eradicates the effect of cisplatin on the swelling‐induced taurine release. As mTORC1 operates downstream to Akt and mTORC2 mediates the growth‐factor‐induced phosphorylation of Akt on Ser‐473 we tested the effect of mTORC1 inhibition (rapamycin) and mTORC2 inactivation (Rictor siRNA silencing). In congruence with data obtained from mouse fibroblasts (Lambert et al. 2014) rapamycin treatment boosts the swelling‐induced taurine release and accelerates inactivation of the release pathway (Figs. 4A–C). Furthermore, the data in Figs 4B and C indicate that the inhibitory effect of cisplatin on the volume‐sensitive taurine release seen in Figures 3E and F is overruled by rapamycin. mTORC2 is, in contrast to mTORC1, insensitive to rapamycin inhibition (Bai et al. 2017) and it is assumed that the contribution of mTOR at a low‐dose of rapamycin allocates from an effect via mTORC1 to an effect via mTORC2. From Figures 4E and F it is seen that inhibition of mTORC2, through Rictor silencing (verified in Fig. 4D), decreases the swelling‐induced taurine release and inactivation of the release pathway. This is similar to the effect of PI3K inhibition (Wortmannin, Figs. 3E and F) and opposite to mTORC1 inhibition (rapamycin, Figs. 4A–C). Based on this, it is suggested that rapamycin induced inhibition of mTORC1, potentiates Akt activation through mTORC2. Hence, the effect of cisplatin on VRAC is partly mediated through modulation of the PI3K/PTEN‐mTORC2‐Akt pathway and intracellular ROS production.

### Correlation between LRRC8A protein overexpression and p53 phosphorylation in response to cell stress

**Figure 5:**
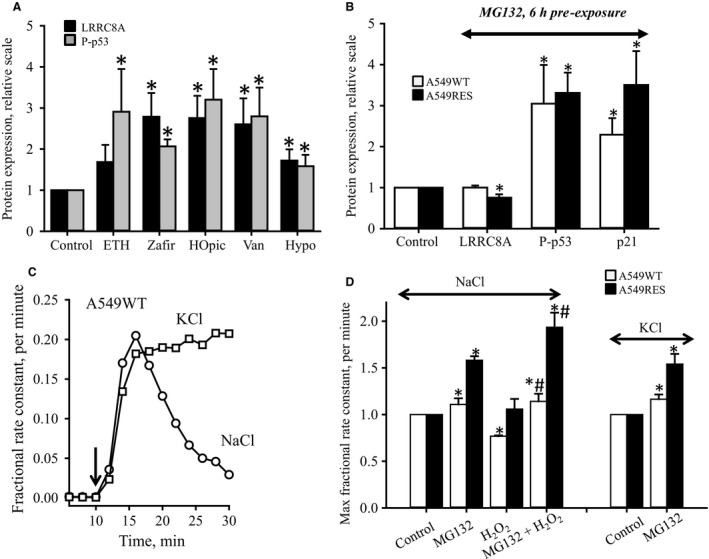
Correlation between LRRC8A expression, VRAC activity and p53 phosphorylation. (A) A549WT cells were incubated for 24 h in isotonic growth medium containing 10 *μ*mol L^−1^ ETH (5‐lipoxygenase inhibitor), 60 *μ*mol L^−1^ Zafirlukast (Zafir, CysLT1 receptor antagonist), 5 *μ*mol L^−1^ HOpic (PTEN inhibitor), 50 *μ*mol L^−1^ Vanadate (Van, phosphatase inhibitor), or hypotonic (Hypo) growth medium. Proteins were extracted and tested for the expression of LRRC8A, phosphorylated p53 (P‐p53) and GAPDH (house holding protein). Protein expression relative to GAPDH was calculated and the increase in protein expression relative to control cells determined. Data shown represent mean values ± SEM from 6 (LRRC8A) and 4 (P‐p53) sets of paired experiments. *indicates that the protein expression in drug‐treated cells was significantly increased from control cells (*P* < 0.05, paired Student′s *t*‐Test). (B) A549WT (open bars) and A549RES (black bars) were preincubated for 6 h in growth medium in the absence or presence of the proteasome inhibitor MG132. Proteins were extracted and tested for expression of LRRC8A, phosphorylated p53 (P‐p53), p21, and GAPDH. Protein expression was in 4 sets of experiments for each cell line normalized to GAPDH and presented relative to the respective nontreated control cells. *indicates that the protein expression was significantly different from control cells (*P* < 0.05, paired Student′s *t*‐Test). (C) Fractional rate constants (min^−1^) for [^3^H]taurine release from A549WT cells in isotonic/hypotonic NaCl or KCl media. In KCl media Na^+^ was substituted by K^+^ to prevent net loss of KCl (RVD) and hence indirect inactivation of the volume‐sensitive taurine release pathway. Time traces represent 5 sets of independent experiments. (D) A549WT (open bars) and A549RES (black bars) cells were preincubated for 6 h in growth medium in the absence (Control) or presence of MG132 (10 *μ*mol L^−1^), H_2_O_2_ (100 *μ*mol L^−1^) or MG132 plus H_2_O_2_ before exposure to isotonic / hypotonic NaCl or KCl media. The fractional rate constants (min^−1^) for [^3^H]‐taurine release was followed with time under isotonic and hypotonic conditions and the maximal rate constant determined as the rate constant at time 6 min after hypotonic exposure as indicated in C. Maximal rate constants are normalized to untreated control cells and presented as the mean values ± SEM. from 4 to 5 sets of experiments. * and # indicate statistical difference between H_2_O_2_/MG132 treated and control cells and between H_2_O_2_ and H_2_O_2_/MG132‐treated cells, respectively, (*P* < 0.05, ANOVA, Fisher LSD Method).

The data in Figures 2B and D show that there is a correlation between LRRC8A expression and activation of p53 following exposure to H_2_O_2_. This prompted us to test whether other forms of cell stress could elicit a similar response. From Figure 5A it is seen that LRRC8A expression as well as p53 phosphorylation are increased following preexposure to pharmacological 5‐LO inhibitors (ETH), CysLT1 receptor‐antagonists (zafirlukast), phosphatase inhibitors (HOpic, Vanadate) as well as hypoosmotic conditions. This indicates that prolonged drug exposure and osmotic perturbation trigger a general cell stress condition resulting in increased LRRC8A protein expression and p53 activation and hence instigation of apoptosis. To determine whether p53 phosphorylation is dictated by increased LRRC8A or whether p53 controls the LRRC8A expression we exposed A549 cells to the cell‐permeable proteasome inhibitor MG132. It has previously been shown that p53 expression in NIH3T3 mouse fibroblasts increased dramatically after addition MG132, which was taken to indicate that p53 levels in unpertubated cells was kept low by proteasomal degradation (Lambert et al. 2015a). From Figure 5B it is seen that 6hs exposure to MG132 increases p53 phosphorylation significantly but has no effect on LRRC8A expression in the cisplatin sensitive A549WT cells. Furthermore, LRRC8A expression is significantly reduced in MG132‐treated A549RES even though p53 activity is high (Fig. 5B). Thus, increased p53 activity does not seem to elicit an increase LRRC8A expression. The increased p21 expression following MG132 exposure probably reflects the concomitant increase in p53 activation. To test the effect of proteasome inhibition of the swelling‐induced taurine release we exposed A549 cells to hypotonic NaCl or KCl media. The KCl medium was chosen in order to prevent net loss of KCl and to prolong the time period where VRAC is maximally active (verified in Fig. 5C). From Figures 5C and D it is seen that MG132 increases the volume‐sensitive taurine release in A549WT and A549RES cells exposed to hypotonic NaCl and KCl media although the LRRC8A protein expression is concomitantly unaltered (A549WT) or reduced (A549RES) (Fig. 5B). The boosting effect on taurine release of proteasome inhibition is seen also in the presence of H_2_O_2_ (Fig. 5D). Hence, proteasome inhibition does not seem to imply regulation of the ROS sensitive elements involved in modulation of VRAC complex activity. The LRRC8A protein itself is prone to oxidation and ubiquitination and hence degradation via the proteasome due to the expression of tyrosine and lysine residues, respectively. Using LRRC8A^−/−^ HEK cells in which we reinstalled vectors coding for native LRRC8A, LRRC8A mutants with tyrosine substituted with phenylalanine or lysine substituted with arginine we followed taurine release as a function of time under isotonic conditions (320 mOsm) and following hypotonic exposure (200 mOsm). The maximal rate constant (min^−1^) for the volume‐sensitive taurine release was determined at 0.014 ± 0.003 (native HEK, *n* = 3), 0.006 ± 0.001 (*n* = 6, LRRC8A^−/−^ HEK), 0.018 ± 0.004 (KO with native LRRC8A, *n* = 6), 0.015 ± 0.002 (KO with LRRC8A: Y→F382, *n* = 6), 0.017 ± 0.004 (KO with LRRC8A: Y→F386, *n* = 6) and 0.012 ± 0.001 (KO with LRRC8A: K→R382, *n* = 6). Although, the taurine release was significantly reduced (*P* = 0.05) following lysine to arginine substitution we also found that the LRRC8A (K→R382) protein expression was significantly reduced to 57 ± 10% (*n* = 3, *P* = 0.03) of the protein expression in KO expressing the native LRRC8A protein. Hence, the sites Y382/Y386 and K501 in LRRC8A do not seem to play a role in posttranslational modulation of LRRC8A and VRAC complex activity.

### Correlation between LRRC8A protein expression in the plasma membrane and VRAC activity

**Figure 6:**
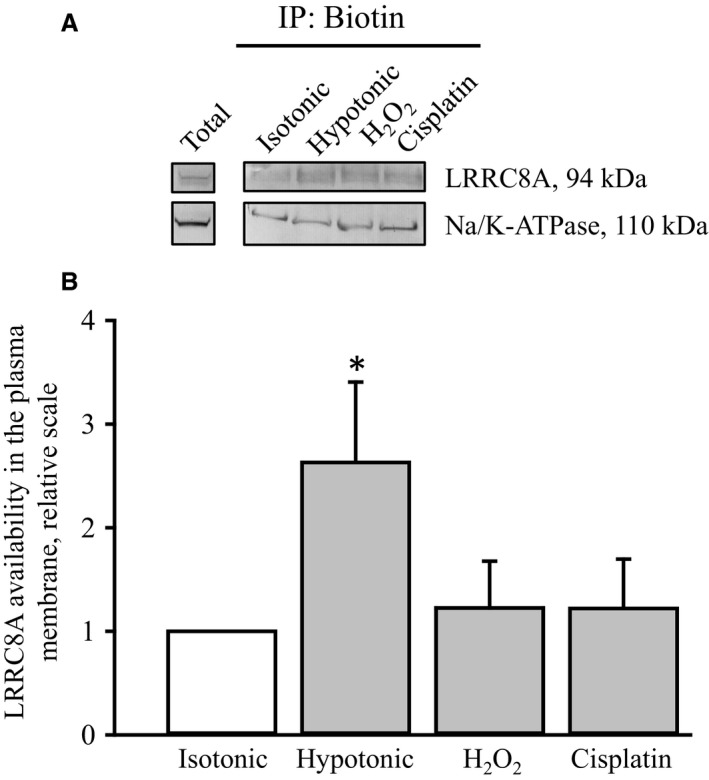
LRRC8A expression in the plasma membrane of A549 cells. Cells were exposed to isotonic conditions (Control), hypotonic conditions and isotonic conditions in the presence of 100 *μ*mol L^−1^ H_2_O_2_ or 10 *μ*mol L^−1^ cisplatin for 18hs. Proteins with components exposed to the extracellular compartment were biotinylated and cells were lysed. Biotinylated LRRC8A and Na^+^/K^+^‐ATPase were extracted and separated/quantified by SDS‐PAGE/western blotting. As the molecular weight of LRRC8A and Na^+^/K^+^‐ATPase are close (94 kDa/110 kDa) western blots were run in parallel on separate gels. (A) Representative blots. Total indicates total cell homogenates prepared from cells not treated with biotin. (B) Protein ratio LRRC8A/Na^+^/K^+^‐ATPase from 3 sets of experiments. *indicates significantly increase compared to isotonic control cells (Student′s *t*‐test).

We have previously demonstrated that reduced VRAC activity in A549 cells with acquired cisplatin resistance correlated with a reduced LRRC8A expression in the plasma membrane even though total LRRC8A protein expression was unaffected or even increased (Sørensen et al. 2016b). From Figure 6 it is seen that long‐term exposure to hypotonic conditions, which increases total LRRC8A expression (Fig. 1C), is accompanied by increased LRRC8A expression in the plasma membrane. On the other hand, the expression of LRRC8A in the plasma membrane is unaffected by long‐term exposure to H_2_O_2_ and cisplatin (Fig. 6) although total levels of LRRC8A protein is increased (Figs 2B and 3D). Hence, reduced VRAC activity following prolonged exposure to hypotonic conditions, ROS and cisplatin, is likely not due to the absence or reduced expression of the indispensable LRRC8A subunit in the VRAC complex.

## Discussion

### Stress‐induced modulation of LRRC8A subunit expression and VRAC complex activity

How cells perceive their volume and how a change in volume is transduced into a volume‐regulatory response has envisioned mechanical deformation/stretch of the plasma membrane and changes in the cytoskeleton or dilution/shift in the intracellular ionic strength through cell swelling/shrinkage and subsequently activation of cell signaling components (phospholipases, lipoxygenases, kinases, phosphatases) involved in the instigation and execution of the volume regulatory process (Pedersen et al. 1999, 2006; Lambert et al. 2006; Hoffmann et al. 2009). An arising question is whether the VRAC complex itself is equipped with a volume sensing capacity. Being the essential component of the VRAC complex a reduction in total LRRC8A protein expression, following exposure to protolichesterinic acid (Thorsteinsdottir et al. 2016), gene silencing (Qiu et al. 2014; Voss et al. 2014) or as a phenotypic characteristic in, for example, cisplatin‐resistant A2780 cells (Sørensen et al. 2014), correlate with a reduced volume‐sensitive taurine release. Likewise, a reduction in LRRC8A protein availability in the plasma membrane, as seen in A549RES compared to A549WT cells (Sørensen et al. 2016b), results in reduced VRAC complex activity, but an unaltered volume set‐point for activation. However, Syeda and coworkers recently showed that LRRC8A (SWELL1), when co‐expressed with other LRRC8 subunits in an artificial bilayer system, was activated by low ionic strength (Syeda et al. 2016). Furthermore, Grodagna and coworkers demonstrated, using expression of fluorescently tagged LRRC8 subunits in *Xenopus* oocytes, that a LRRC8A/8D channel complex was inhibited by oxidation (Gradogna et al. 2017). Although, we find that mutation of preserved tyrosines in LRRC8A (Tyr382, Tyr386) does not reduce volume‐sensitive activation, one cannot exclude that the reduction in volume‐sensitive taurine release, following H_2_O_2_ exposure during hypotonically adaptation, could partly imply oxidation and inactivation of the LRRC8D component in the VRAC complex. A surprising observation was that overexpression of LRRC8A in HEK293T and HeLa cells was not accompanied by an in increase in I_Cl,Swell_ but rather a decrease compared to wild‐type levels (Qiu et al. 2014; Voss et al. 2014). Likewise, we observe that an increased total LRRC8A expression, following hypotonic adaptation (A549) and exposure to ROS and cisplatin (A2780) (Sørensen et al. 2014, 2016a) correlates with reduction in volume‐sensitive taurine release. Our data indicate that LRRC8A mRNA accumulation is reduced after hypotonic adaptation, which probably reflects negative feedback from prolonged hypotonic conditions and / or an increased LRRC8A protein expression. It is noted that the increased LRRC8A availability in the plasma membrane, as seen in hypotonically adapted A549 cells, could explain the concomitant stronger correlation between medium osmolarity and maximal rate constant for volume sensitive taurine release, that is, VRAC activity. Currently we assume that modulation of osmolyte transport via the VRAC complex is mainly controlled through (1) posttranslational modulation of LRRC8 subunits, (2) shift in the stoichiometry between LRRC8A and other LRRC8 family members, (3) modulation of the number of functional VRAC complexes in the plasma membrane, and (4) shift in the activity of signaling cascades involved in modulation of VRAC complex activity.

### ROS mediated modulation of VRAC activity

ROS production increases during physiological stress, for example, cell swelling, muscle contraction sepsis, and anoxia (Lipton 1999; Nethery et al. 1999; Lambert 2003, 2007; Ørtenblad et al. 2003; Varela et al. 2004; Diaz‐Elizondo et al. 2006; Friis et al. 2008; Holm et al. 2013). In the case of A549 cells it has previously been demonstrated that swelling‐induced taurine release is potentiated following acute exposure to ROS but inhibited in the presence of the NADPH oxidase inhibitor DPI (diphenyleneiodonium) as well as the antioxidant BHT (Holm et al. 2013). As A549 increase their ROS production within the first minutes following hypotonic exposure it is assumed that ROS could have an autocrine effect and promote release of organic osmolytes via the VRAC complex. The 5‐LO activity is controlled by ROS and as 5‐LO, by pharmacological and substrate inhibition, has been shown to be required for swelling‐induced activation of taurine in a variety of cell types (Hall 1995; Lambert and Falktoft 2001; Lambert et al. 2001; Lambert 2003; Ørtenblad et al. 2003; Holm et al. 2013; Sørensen et al. 2014) the 5‐LO could constitute a primary target for ROS. In the present study, it is demonstrated that the boosting effect of acute exposure to ROS on the swelling‐induced taurine release similar to the effect of the phosphatase inhibitor vanadate is weakened in hypotonically adapted cells and even reversed into an inhibition in cells exposed to excess H_2_O_2_ during the hypotonic adaptation period. Protein tyrosine phosphatases are inhibited by ROS via oxidation of a cysteine in the catalytic site (Huyer et al. 1997; Meng et al. 2002) and as our experiments indicate that LRRC8A availability in the plasma membrane is unaffected by preexposure to H_2_O_2_ in A549 cells it is assumed that the prolonged ROS exposure reduces VRAC activity due to a shift in the protein tyrosine phosphorylation status of the VRAC complex and/or cellular components involved in the activation of the VRAC complex. It has previously been shown in NIH3T3 mouse fibroblasts that the boosting effect of ROS and vanadate is impaired in the presence of the broad‐spectrum tyrosine kinase inhibitor Genistein (Lambert 2003) implying that the shift in the phosphorylation status could reflect shift in the activity of protein tyrosine kinases. A variety of protein tyrosine kinases, for example, epidermal growth factor receptor, insulin receptor, Src, and Lck, are known to be regulated by redox in cells (Kamata and Hirata 1999) and it has been shown that protein tyrosine phosphorylation occurs concomitant to activation of *I*_Cl,Swell_ (Davis et al. 2001) and taurine efflux (Ochoa de la Paz et al. 2002) in a process that in some occasions involves the focal adhesion kinase (FAK, p125FAK) (Tilly et al. 1996; Ochoa de la Paz et al. 2002). Auto phosphorylation of FAK, elicited by its binding to *β*1‐integrin, facilitates formation of a FAK – Src protein tyrosine kinase complex (Ben Mahdi et al. 2000) and subsequently phosphorylation of FAK in its catalytic and C‐terminal domains (Salazar and Rozengurt 1999; Ben Mahdi et al. 2000). An elevated phosphorylation at Tyr576/Tyr577 in the catalytic domain of FAK, detected in vanadate treated cells overexpressing chicken FAK, is previously demonstrated to be essential for an increase in FAK kinase activity (Maa and Leu 1998). In congruence H_2_O_2_ has been shown to induce a time‐dependent phosphorylation of FAK in bovine pulmonary artery endothelial cells, which occurs primarily through inhibition of protein tyrosine phosphatases, specific to FAK (Vepa et al. 1999). In this context it is noted that the volume‐sensitive taurine release in NIH3T3 cells has been shown to be reduced following inhibition of kinase activity coupled to the epidermal growth factor (EGF) receptor and boosted by application of EGF (Lambert 2003), whereas the volume‐sensitive taurine release in A549 is inhibited by the tyrosine kinase inhibitor Genistein and the selective Janus kinase (JAK) inhibitor cucurbitacin (Holm et al. 2013). Whether adaptation in A549 cells to excess ROS involves shift in the expression/activity of specific protein tyrosine receptor kinases was not investigated in the present study.

### LRRC8A protein expression and apoptosis

Our present data indicate that exposure to ROS (H_2_O_2_) as well as cisplatin induces an increase in LRRC8A expression, Akt activity, p53 phosphorylation and subsequently apoptosis and presumably inhibition of cell cycle progression (increase in p21). Reduction in cell volume, due to net loss of ions (KCl), organic osmolytes (taurine and other nonessential amino acids) and cell water is a hallmark of AVD and it has been demonstrated that cell shrinkage per se instigates apoptosis through induction of protein kinase p38 and p53 mediated signaling, stimulation of death receptor (CD95) trafficking to the plasma membrane or impairment of growth factor‐receptor‐mediated signaling (Hoffmann et al. 2009). In recent years it has turned out that cisplatin resistance in cancer cells can reflect down‐regulation in the activity of volume‐sensitive channels for K^+^, Cl^−^ and taurine (Lee et al. 2007; Poulsen et al. 2010; Min et al. 2011; Hoffmann and Lambert 2014; Sørensen et al. 2014), which seems to limit or postpone apoptosis. It is noted that restriction of taurine loss via the VRAC complex as seen in, for example, cisplatin‐resistant A2780 cells (Sørensen et al. 2014) might be envisaged as a survival strategy, as taurine besides being a quantitative important osmolyte, also has been indicated to reduce progression of the apoptotic process following cell stress, for example, drug exposure, ischemia/hypoxia and glucose supplementation, through prevention of (1) mitochondrial dysfunction (2) upregulation of proapoptotic messengers (Bax, Fas), (3) p53 mediated activity, (4) ROS generation, (5) Ca^2+^ mobilization, (6) apoptosome assembly, and caspase activity (Lambert et al. 2015b). In the context of brain physiology, it has to be emphasized that taurine activates inhibitory glycine/*γ*‐aminobutyric acid receptors (Le‐Corronc et al. 2011) and that limitation of taurine release during ischemia‐ / hypoxia‐induced cell swelling might affect neurotransmission. We have previously shown that (1) LRRC8A expression, hence activation of the LRRC8A and presumably osmolyte transport via a VRAC complex, is essential for cisplatin‐induced instigation of the proapoptotic transcription factor p53 and its down‐stream signaling (caspase‐9/‐3 activation, expression of p21Waf1/Cip1, MDM2), (2) down‐regulation of LRRC8A‐dependent transport activity contributes to acquirement of cisplatin resistance in ovarian and lung carcinoma cells, and (3) that activation of the LRRC8A‐dependent channels is upstream to the cisplatin‐induced AVD as apoptosis induced by hypertonic cell shrinkage does not require the presence of LRRC8A/VRAC (Sørensen et al. 2016b). Our present findings indicate that prolonged treatment of A549 cells with ROS or inhibitors/receptor antagonists, that are normally used to verify a role of element in the volume signaling cascade, actually increases LRRC8A protein expression and concomitantly phosphorylation/activation of p53. In the case of H_2_O_2_ the exposure is followed by instigation of apoptosis, cell cycle arrest as well as a reduction in volume‐sensitive taurine release. The latter could reflect an attempt to limit AVD and the apoptotic process. As was the case with cisplatin‐induced apoptosis the upregulated p53 activity is downstream to the concomitant upregulation of LRRC8A, as an increase in p53 phosphorylation per se, provoked by the presence of the proteasome inhibitor MG132, is not accompanied by an increase in LRRC8A. We have previously demonstrated that a low p53 expression in unperturbed NIH3T3 mouse fibroblasts reflects a balance between high syntheses of p53 and an equally high proteasome breakdown of p53 which is prevented by the addition of MG132 (Lambert et al. 2015a). It is noted that inhibition of proteasome activity provokes an amplification of the swelling‐induced taurine release and presumably apoptosis and cell cycle arrest (increased p53 phosphorylation, p21 expression). The effect of proteasome inhibition does not seem to imply ubiquitination and proteomic removal of LRRC8A, as mutation of a putative ubiquitination site on LRRC8A diminished LRRC8A expression and VRAC complex activity to the same extent.

FoxO3a phosphorylation in A549 cells decreases during prolonged H_2_O_2_ exposure concomitantly to an increase in Akt activity, which could indicate that Akt mediates nuclear export and inactivation of FoxO3. FoxO3 mediates transcriptional regulation of the ubiquitin ligase adaptor Keap1 (Kelch‐like ECH‐associated protein 1) which targets the nuclear factor Nrf2 (Nuclear factor (erythroid‐derived 2)‐like 2) for ubiquitination and proteasomal degradation. As Nrf2 is a proto‐oncogene, that protects cells against cell damage and apoptosis through transcriptional regulation of detoxifying enzymes and anti‐apoptotic members (Bcl‐2, Bcl‐XL), it is assumed that increased Akt activity, following cisplatin and ROS exposure, results in an increased expression of Nrf2 and its down‐stream anti‐apoptotic genes. Akt also activates the murine double minute‐2 (MDM2), which negatively regulates p53 activity (Steelman et al. 2011). As p53 suppress the Nrf2 promoter activity by blocking Sp1 binding to the Nrf2 promoter an increased Akt activity will not only limit p53 mediated activation of apoptosis and cell cycle arrest but also limit p53 mediated inhibition of Nrf2. Hence, Akt will induce cell survival.

### Cell stress and Akt‐mTORC1/‐mTORC2 signaling

The serine/threonine kinase mTOR is a well‐characterized substrate of Akt and it is known that Akt‐mediated activation of mTOR occurs through phosphorylation and inactivation of tuberous sclerosis complex 2 (TSC2), which normally inhibits mTOR (Hay 2005). mTOR constitutes the catalytic unit of mTORC1, which is distinguished from mTORC2 by having Raptor as catalytic regulator. In contrast to mTORC1, that acts down‐stream to Akt, mTORC2 seems to be involved in phosphorylation (Ser‐473) and activation of Akt in response to stimulation of receptor‐tyrosine kinases (Dasari and Tchounwou 2014). Binding of the translation initiation factor 4E‐BP1 and the kinase p70‐S6 to Raptor is required for mTORC1 mediated phosphorylation of 4E‐BP1 and facilitation of cap‐dependent translation. Rapamycin forms a complex with its cellular receptor FKBP12 that subsequently disrupts Raptor and mTOR interaction and hence prevent mTORC1 activation. It has been demonstrated in epidermal cells that a low‐dose of rapamycin potentiated the mTORC2 pathway, that is, increased SIN‐1 (Thr‐86) and Akt (Ser‐473) phosphorylation (Bai et al. 2017). mTORC2, that contains the catalytic regulator Rictor, has opposing functions compared to mTORC1 (Gonzalez and Rallis 2017) and it has turned out that mTORC2‐mediated phosphorylation and activation of Akt seem to be required for cell cycle progression in cancer cells (Hietakangas and Cohen 2008). We have previously shown that mTORC1 activity, detected as phosphorylation of 4E‐BP1, is transiently increased in mouse fibroblasts following hypoosmotic exposure, that is, mTOR activity is significantly increased and boosted by PTEN inhibition within minutes following osmotic cell swelling but reduced following prolonged (4 h/24 h) hypotonic adaptation (Lambert et al. 2014). Using rapamycin, to mimic the effect of reduced mTORC1 activity, during prolonged hypotonic conditions it was shown that reduced mTORC1 activity in the mouse fibroblasts correlated with a reduce catalase mRNA accumulation (Lambert et al. 2014). As catalase inhibition, similar to rapamycin inhibition, boosted the volume‐sensitive taurine release in the mouse fibroblasts it was suggested that chronic hypotonic exposure and mTORC1 inhibition reduce the antioxidative defense (Lambert et al. 2014). Whether a reduced ability to remove ROS contributes to the reduced ROS sensitivity during hypotonic adaptation can at the moment only be speculative.

In human lung A549 cancer cells Akt‐1 amplification was found to promote cisplatin resistance through the mTORC1/p70S6K‐1 signaling pathway (Liu et al. 2007). We find that albeit cisplatin increases Akt phosphorylation /activity as well as LRRC8A protein expression in A549 it concomitantly reduces VRAC complex activity. Inhibition of PI3K activity by addition of wortmannin prevents Akt activation and at the same time eradicates the effect of cisplatin on Akt phosphorylation and VRAC activity, that is, the effect of cisplatin on VRAC involves PI3K‐Akt signaling. Inhibition of mTORC1 with rapamycin increases Akt phosphorylation but prevents the cisplatin‐induced reduction in VRAC activity. In contrast, inhibition of mTORC2 through Rictor silencing reduces similar to PI3K inhibition swelling‐induced VRAC activation. mTORC2 is insensitive to rapamycin inhibition (Bai et al. 2017) and it is assumed that the contribution of mTOR at a low‐dose of rapamycin allocates from an effect via mTORC1 to an effect via mTORC2, that is, inhibition of mTORC1, potentiates mTORC2‐mediated Akt activation. Hence, the effect of cisplatin on VRAC is partly mediated through modulation of the PI3K/PTEN‐mTORC2‐Akt pathway and intracellular ROS production.

**Figure 7:**
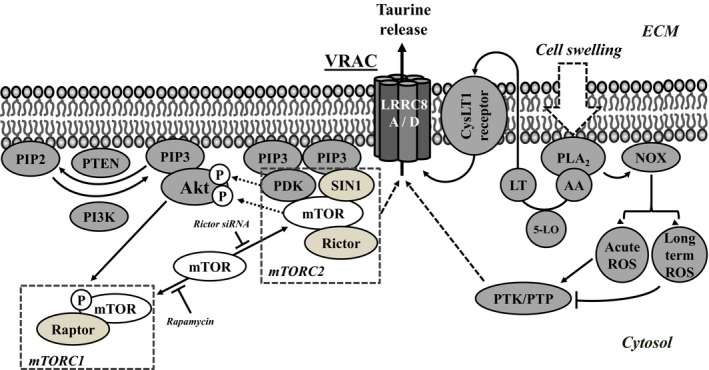
Activation and stress modulation of cell signaling pathways involved in release of taurine through the VRAC complex in human lung epithelial cell line A549. Volume‐sensitive release of taurine through VRAC, with a presumable LRRC8A/D stoichiometry, requires phospholipase A_2_, 5‐LO and CysLT1 activity. The VRAC activity is attenuated by the CysLT1 receptor antagonist Zafirlukast and the 5‐lipoxygenase inhibitor ETH. Cell swelling generates ROS via the NADPH oxidase (NOX) system, which either potentiates (acute osmotic exposure) or inhibits (long‐term adaptation) swelling‐induced taurine release. The ROS induced regulation on VRAC activity is believed to involve increased net tyrosine‐phosphorylation of elements, involved in the volume‐sensitive signaling cascade. The role of endogenous ROS is mimicked by addition of H_2_O_2_ and vanadate. Cell growth factor‐dependent modulation of VRAC involves the PI3K‐Akt‐mTOR signaling cascade. BpV(HOpic) and Wortmannin inhibit PTEN and PI3K, respectively. mTOR activity depends on its recruitment to mTORC1 or mTORC2 (dotted squares). Rapamycin disrupts mTORC1 complex formation and thereby enhances mTOR the chance of mTORC2 complex formation. Rictor siRNA prevents mTORC2‐mediated activity. Cisplatin attenuates release of taurine though Akt‐mediated signaling. VRAC, volume‐regulated anion channel.

Figure 7 highlights elements in the intracellular signaling provoked by cell swelling (PLA_2_‐5LO‐CysLT1) and modulated by ROS (NADH‐oxidases, protein tyrosine kinases and phosphatases) or cell growth factors (PI3K‐Akt‐mTOR). In the present work, we demonstrate that inhibition of mTORC1 with rapamycin, similar to acute H_2_O_2_ exposure, boosts swelling‐induced taurine release from A549 cells, whereas Rictor knockdown significantly reduced the release to the same level as achieved by treatment with the PI3K inhibitor wortmannin. Signaling by mTORC2 seems to boost volume‐sensitive taurine release in A549 cells and our data are taken to indicate that inhibition of mTORC1 with rapamycin, favors mTOR complex 2 formation and hence taurine release. Our current hypothesis is that although prolonged exposure to excess ROS and cisplatin instigates apoptosis and cell cycle arrest in human alveolar carcinoma cells (A549) a concomitant increase in LRRC8A protein expression and Akt activation seems to reduce VRAC complex activity which would limit loss of osmolytes (AVD) and hence progression of apoptosis.

Since the discovery that LRRCA was essential for VRAC complex activity it has turned out that LRRC8A is critical for a variety of physiological function, for example, T‐cell development (Kumar et al. 2014), cell proliferation (Rubino et al. 2018) and adipogenesis (Zhang et al. 2017). In the latter case it was indicated that during adipocyte hypertrophy the increase in cell volume will activate LRRC8A and potentiate intracellular insulin signaling and hence boost GLUT4 translocation to the plasma membrane, glucose uptake as well as lipogenesis (Zhang et al. 2017). Furthermore, Zhang and coworkers also demonstrated that LRRC8A (SWELL1) knock out in adipocytes reduced insulin‐PI3K‐pAKT2 stimulated GLUT4 translocation and that LRRC8A knock down *in vivo* was associated with an impaired glucose tolerance as well as insulin resistance (Zhang et al. 2017). Our findings indicate that cell‐stress, for example , prolonged exposure to various drugs, ROS, and anisotonic conditions, reduces VRAC activity in human alveolar carcinoma cells even though the expression of the essential VRAC component LRRC8A is concomitantly increased. During treatment of cancer cells with cisplatin a reduced VRAC activity will limit accumulation of the Pt‐based drug and hence instigation of apoptotic cell death.

## Conflict of Interest

None declared.
